# Alimentary Canal of the Adult Blow Fly, *Chrysomya megacephala* (F.) (Diptera: Calliphoridae)—Part I: Ultrastructure of Salivary Glands

**DOI:** 10.1155/2012/382917

**Published:** 2012-05-13

**Authors:** Worachote Boonsriwong, Kabkaew L. Sukontason, Tarinee Chaiwong, Urai Chaisri, Roy C. Vogtsberger, Kom Sukontason

**Affiliations:** ^1^Faculty of Allied Health Science, Burapha University, Chonburi 20131, Thailand; ^2^Department of Parasitology, Faculty of Medicine, Chiang Mai University, Chiang Mai 50200, Thailand; ^3^College of Medicine and Public Health, Ubon Ratchathani University, Ubon Ratchathani 34190, Thailand; ^4^Department of Tropical Pathology, Faculty of Tropical Medicine, Mahidol University, Bangkok 10400, Thailand; ^5^Department of Biology, Midwestern State University, Wichita Falls, TX 76308, USA

## Abstract

The salivary gland ultrastructure of the adult male blow fly, *Chrysomya megacephala* (F.) (Diptera: Calliphoridae), was investigated at the ultrastructural level using light microscopy (LM), scanning electron microscopy (SEM), and transmission electron microscopy (TEM). The salivary glands are paired structures composed of a single median deferent duct bifurcated into two long, narrow efferent ducts connected to the coiled tubular glands. The SEM image of the gland surface revealed that the basal lamina is relatively smooth in general, but the whole surface appeared as a trace of rough swollen insertion by intense tracheal ramification. Ultrastructurally, the salivary gland is enclosed within the basal lamina, and interdigitation cytoplasmic extensions were apparent between the adjacent gland cells. The basement membrane appeared infoldings that is similar to the complex of the labyrinth channel. The cytoplasm characteristic of the gland revealed high activity, based on the abundance of noticeable secretory granules, either singly or in an aggregated reservoir. In addition, mitochondria were found to intersperse among rich parallel of arrays rough endoplasmic reticulum. Thick cuticle, which was well-delineated and electron dense, apically lined the gland compartments, with discontinuity of the double-layer cuticle revealing a trace of secretion discharged into the lumen. Gross anatomy of the adult salivary gland was markedly different from that of the third instar of the same species, and structural dissimilarity is discussed briefly.

## 1. Introduction

*Chrysomya megacephala* (F.), or the Oriental latrine fly, is a medically important blow fly species. Its adults are not only annoying to humans and animals, but they also act as a potential mechanical disseminator of pathogens that may cause diseases [1, 2]. In some Southeast Asian countries, adult flies cause damage in fermented fish when females oviposit on this product, resulting in infestation of fly larvae [3]. Furthermore, myiasis produced by the larvae of this fly has been reported increasingly in human cases [4–6]. Geographically, *C. megacephala* is distributed widely over continents worldwide, extending from Oriental Asia, Australasia, Africa, Europe, the Mediterranean to North and South America [7–9]. In northern Thailand, systematic surveys revealed that* C. megacephala* is the most common species collected in many habitats, ranging from urban human to rural and forest environments, from which the number of *C. megacephala* collected was more than that for the house fly, *Musca domestica* [10].

 Based on the close association of *C. megacephala* with humans and/or animals, which may be either disadvantageous or desirable from a forensic entomology viewpoint, diverse biological knowledge pertaining to this fly is essential in order to manage it. Morphological information on both the gross and ultrastructural level is not exempt in this regard. With respect to the various internal systems of flies, the alimentary one is accountable for successful feeding and it dislodges unused food materials; therefore, ultrastructure of the alimentary canal has been investigated intensively in several species of insects and flies. Examples of this were recorded in the salivary glands of *Calliphora erythrocephala* (Diptera: Calliphoridae) [11], *Dermatobia hominis* (Diptera: Oestridae) [12], *Heliothrips haemorrhoidalis* (Thysanoptera: Thripidae) [13], *Atta sexdens rubropilosa* (Hymenoptera: Formicidae) [14], *Cimex hemipterus* [15], *Triatoma infestans* [16],* Mahanarva fimbriolata* [17], and *Ixodes ricinus* (Acari: Ixodidae) [18], or the midgut of *D. hominis* [19], *Ceroplastes japonicus* (Hemiptera: Coccidae) [20], and *Belgica Antarctica* (Diptera: Chironomidae) [21]. Ultrastructure of the salivary glands should be researched increasingly, based on verification of salivary gland hypertrophy viruses (SGHVs), which are entomopathogenic and induce salivary gland hypertrophy in dipteran hosts [22]. Recently, two hytrosaviruses, MdSGHV and GpSGHV, were found to induce distinct cytopathology in the salivary glands of *M. domestica* and the tsetse fly, *Glossina pallidipes*, respectively [23], causing pathological symptoms after infection. Thereby, MdSGHV may have potential in a management strategy for house fly populations [24]. As for *C. megacephala*, the alimentary canal ultrastructure of the third instar was documented [25], but information pertaining to the salivary gland in its adult is lacking. Therefore, this study aimed to investigate the salivary gland of adult male *C. megacephala* at the ultrastructural level using light microscopy (LM), scanning electron microscopy (SEM), and transmission electron microscopy (TEM) to provide relevant baseline information. Special attention has been placed on both gross anatomy and cellular structure of this organ.

## 2. Materials and Methods

### 2.1. Rearing of *C. megacephala*

Flies were collected from local marketplaces in Chiang Mai, northern Thailand, and subsequently reared. This fly colony was maintained at ambient temperature (18–27°C), with a natural light/dark photoperiod, in a cabinet in the rearing room of the Department of Parasitology, Faculty of Medicine, Chiang Mai University. Adults were reared on two kinds of food: (i) a mixture of 10% (w/v) multivitamin syrup solution and (ii) fresh pork liver (used as both a food source and oviposition site), while larvae were provided with fresh pork liver.

### 2.2. Dissection of the Salivary Gland

Dissection of 7-day-old males was performed in a phosphate buffer solution (pH = 7.4) using two fine forceps under a binocular dissecting microscope (Olympus, Japan). Photographs were taken using an Olympus C4040Z digital camera (Olympus, Japan). The protocol of dissection has been described previously [26].

### 2.3. Scanning Electron Microscopy (SEM)

The dissected salivary gland was processed for the SEM study, with the procedure being recorded [25]. The micrographs were viewed with a JEOL JSM-5910LV scanning electron microscope (JEOL, Japan).

### 2.4. Transmission Electron Microscopy (TEM)

 Preparation of the salivary gland specimens was processed, with the protocol being the same as that for the third instar [25]. Ultrathin sections (90 nm) were cut and stained with uranyl acetate and lead citrate before being observed in a Hitachi H700 transmission electron microscope (Japan) operated at 100 kV.

## 3. Results

**Figure 1:**
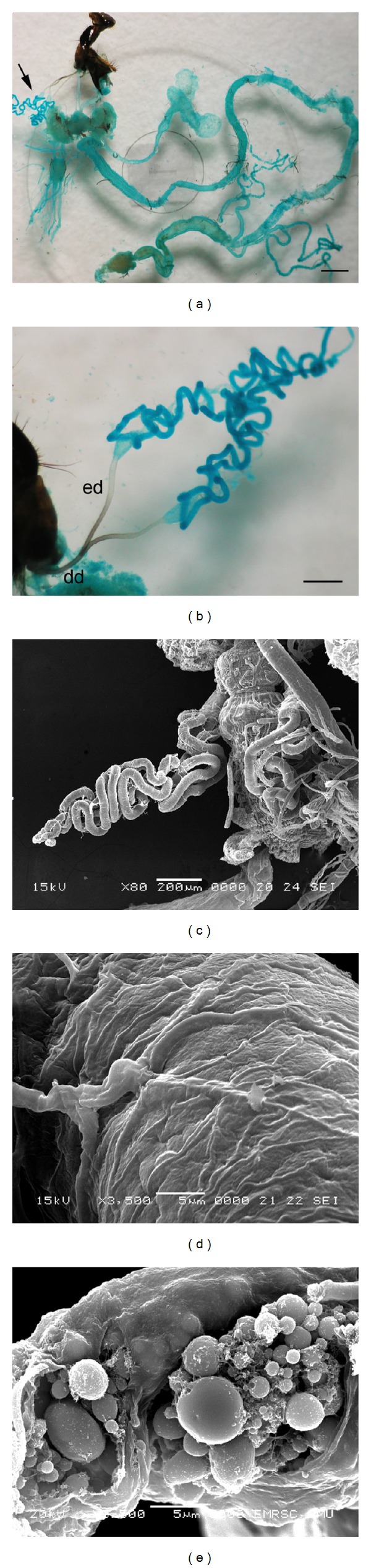
Micrographs of the salivary gland in adult male *C. megacephala*. (a) Light micrograph of the whole excised alimentary canal showing salivary glands (arrow) situated in the foregut region. Bar = 10 *μ*m. (b) Light micrograph indicating a translucent single median deferent duct (dd) bifurcated into two long, narrow efferent ducts (ed) connected to the coiled tubular glands. Bar = 5 *μ*m. (c–e; SEM micrographs) (c) Coil salivary gland at one side. (d) Intense tracheal ramification onto the gland surface. (e) Ruptured gland exhibiting numerous rounded vesicles that are probably secretory granules.

Observation of the whole excised alimentary canal of male *C. megacephala* under LM demonstrated that the salivary glands were a paired structure situated in the foregut region (Figure 1(a)). The gland comprised a translucent single median deferent duct was inserted into the mouthpart at the junction between the rostrum and haustellum (Figures 1(a) and 1(b)). Two long and narrow efferent ducts, bifurcated from the deferent duct, were connected to the long narrow coiled tubular glands (Figures 1(b) and 1(c)). The SEM image of the gland surface revealed that the basal lamina was relatively smooth in general, but the whole surface appeared as a trace of rough swollen insertion by intense tracheal ramification (Figure 1(d)). A ruptured gland in another SEM image exhibited numerous rounded vesicles inside the gland, which were probably secretory granules of variable sizes (Figure 1(d)).

**Figure 2:**
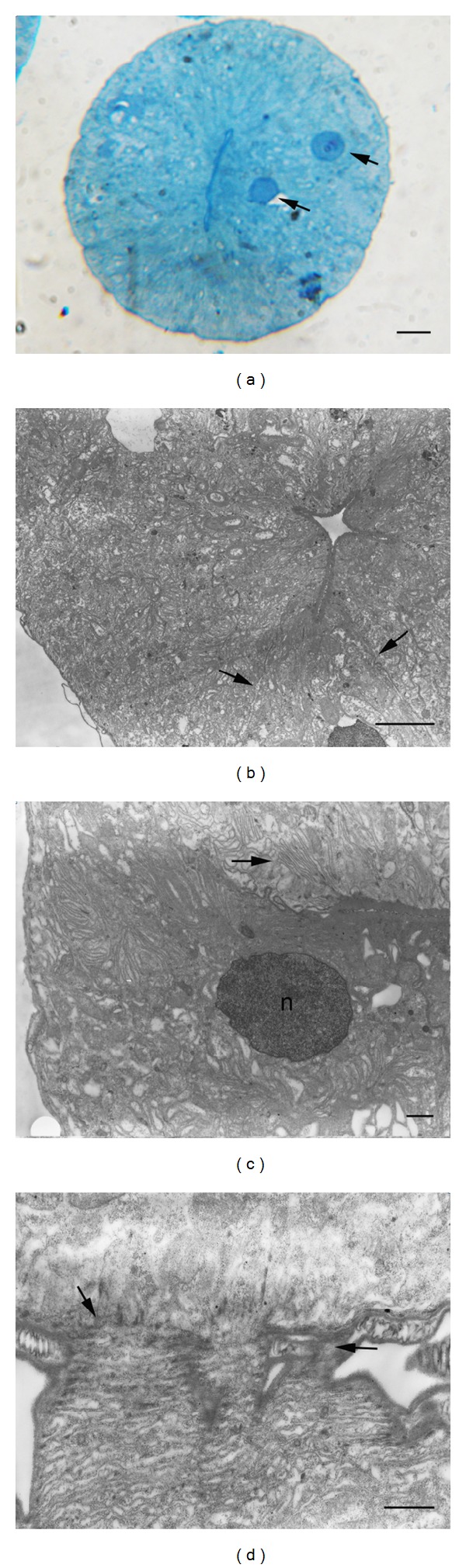
Micrographs of the salivary gland in adult male *C. megacephala*. (a) Light micrograph of the thick section showing the narrow central gland lumen. Arrows indicate a large nucleus. Bar = 5 *μ*m. (b–d; TEM micrographs) (b) Compartment of the gland context (arrows). Bar = 5 *μ*m. (c) Baso-lateral region of the gland cell, exhibiting infoldings of the basement membrane (arrow). A large nucleus (n) is observed. Bar = 1 *μ*m. (d) Interdigitation of cytoplasmic extensions between the adjacent gland cells (arrows). Bar = 1 *μ*m.

The salivary gland revealed a rounded structure in the thick section that was enclosed within the basal lamina. The narrow gland lumen was situated centrally, with an apparently large nucleus (Figure 2(a)). The gland structure was more noticeable under TEM images, illustrating the compartment of the gland context (Figures 2(b) and 2(c)). Observation under higher magnification illustrated that the basolateral region of the gland cell exhibited infoldings of the basement membrane (Figure 2(c)). Images also suggested interdigitation cytoplasmic extensions between adjacent gland cells (Figure 2(d)).

**Figure 3:**
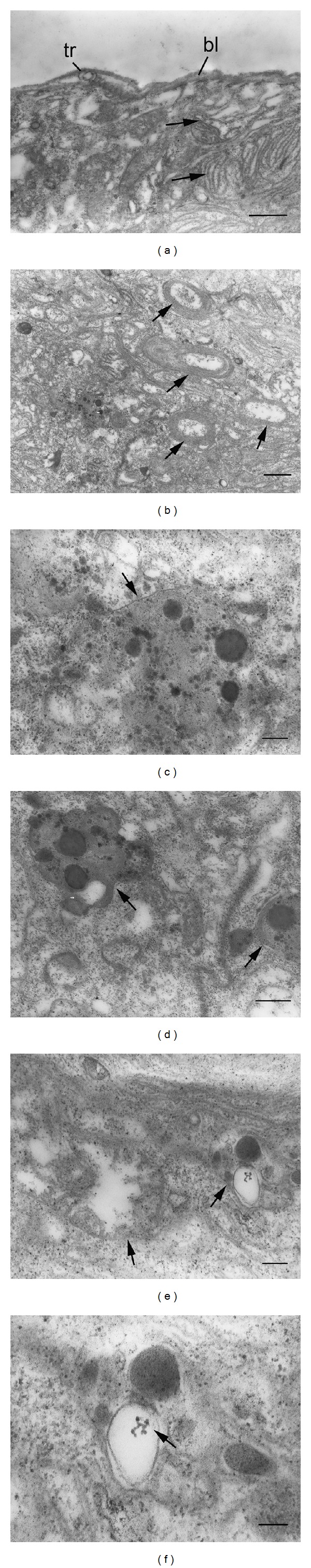
TEM micrographs of the salivary gland in adult male *C. megacephala*. (a) Salivary gland cells highlighting the basal lamina (bl), and infoldings of the basement membrane (arrows). Evidence of small tracheole (tr) insertion. Bar = 1 *μ*m. (b) The gland cell cytoplasm showing aggregation of the oval reservoir of secretory materials (arrows). Bar = 1 *μ*m. (c) Secretory reservoirs containing electron dense secretory granules (arrow). Bar = 2 *μ*m. (d) Aggregation of secretory reservoirs (arrows). Bar = 0.5 *μ*m. (e) Variable secretory reservoir (arrows). Bar = 0.2 *μ*m. (f) Secretory reservoir demonstrating globulin-like material (arrow). Bar = 1 *μ*m.

**Figure 4:**
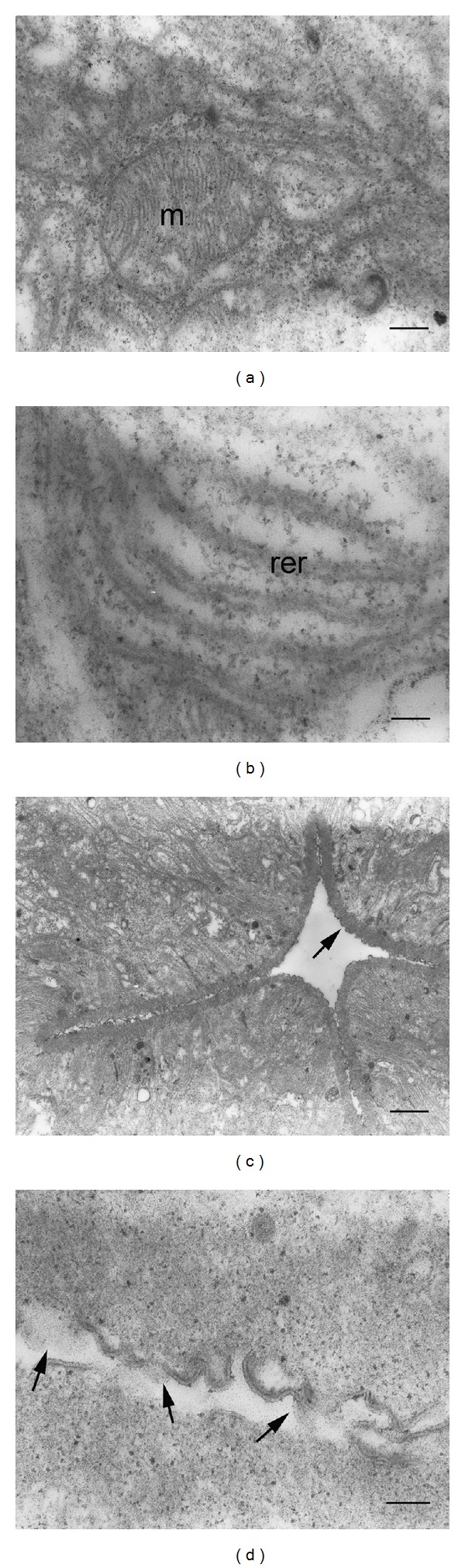
TEM micrographs of the salivary gland in adult male *C. megacephala*. (a) Salivary gland cell presenting mitochondria (m). Bar = 0.2 *μ*m. (b) Parallel arrays rough endoplasmic reticulum (rer). Bar = 0.1 *μ*m. (c) Gland lumen indicating an electron dense thick cuticle (arrow). Bar = 1 *μ*m. (d) High magnification of a cuticle exhibiting trace of secretory discharge into the lumen (arrows). Bar = 0.1 *μ*m.

Ultrastructurally, the outermost part of the gland was outlined with thin basal lamina, inserted with small tracheoles (Figure 3(a)). The basement membrane was very close to the basal lamina and appeared as infoldings similar to the complex of the labyrinth channel (Figure 3(a)). The gland cell cytoplasm was rich with aggregation of variable electron dense secretory granules (Figures 3(b)–3(f)), some of which had the oval structure of secretory materials wrapped in several thin layers (Figure 3(b)). A cluster of reservoirs containing electron dense secretory granules was prominent (Figures 3(c)–3(f)), while globulin-like material, similar to protein synthesis, was observed in the reservoirs (Figure 3(f)). In addition, the gland cell cytoplasm presented mitochondria (Figure 4(a)) interspersed among rich parallel arrays rough endoplasmic reticulum (Figure 4(b)). When highlighting the gland lumen, infoldings of the basement membrane in each gland compartment tended to orient in the gland lumen. The well-delineated electron density of the thick cuticle apically lined the gland compartments (Figure 4(c)). Close-up investigation of the cuticle facing the lumen exhibited a double-layer of discontinuity, which most likely represented a trace of secretion discharged from the gland into the lumen space (Figure 4(d)).

## 4. Discussion

**Figure 5:**
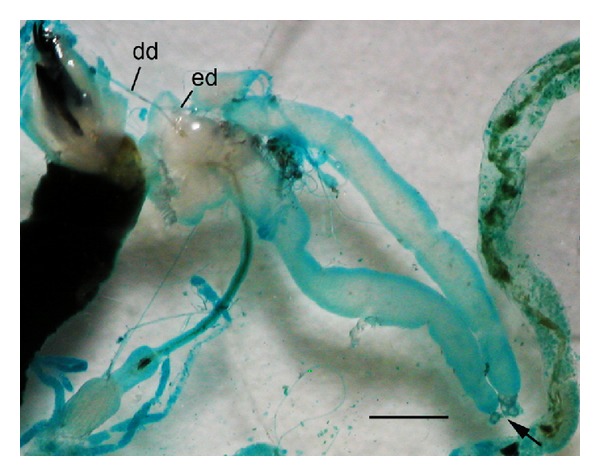
Light micrograph of the salivary gland in third instar *C. megacephala* revealing a translucent single median deferent duct (dd) bifurcated into two long, narrow efferent ducts (ed) terminally connected to the large tubular glands (arrow). Bar = 10 *μ*m.

This study presented morphology of the salivary gland in male *C. megacephala* at the ultrastructural level. Gross morphology of the salivary gland in adult male *C. megacephala* appears as long narrow coiled tubular glands, which are similar to those in females of the same species (data not shown). However, these glands are different in appearance from those already demonstrated in the third instar, which comprise a large long tube (Figure 5) [25]. This information suggests that much anatomical transformation occurs during metamorphosis from larva to adult. Ultrastructural observation of the salivary gland in adult *C. megacephala* presented a thick cuticle layer lined along the apical border of the gland; however, no cuticle was apparent in the third instar [25]. This phenomenon is similar to that found in *Ceratitis capitata* (Diptera: Tephritidae) [27], of which Riparbelli et al. [27] suggested that the larval glands are histolyzed during metamorphosis and the adult glands form imaginal cells situated at the junction between the gland and duct. This transformation reflects adaptation to difference in feeding behavior between larvae and adults, for example, type of food source, food nutrient, or physiological process, as previously described in *D. hominis* [19].

The gross anatomy of salivary gland observed in *C. megacephala* was greatly similar to the illustrated micrograph in adult *M. domestica* [22]. Interestingly, the results under TEM observation revealed doubt in musculature in the outermost part of the salivary gland in male *C. megacephala*. This phenomenon was related to the absence described in *M. domestica *[23] or in the salivary duct of the female scorpion fly, *Panorpa obtusa* [28]. The membrane infoldings of salivary gland cells was observed as analogous to the complex of the labyrinth channel, in accordance with investigations in *C. capitata* [27],* C. hemipterous* [15], or even mouse [29]. Similarly, the rough endoplasmic reticulum, which is often arranged in parallel arrays observed in male *C. megacephala,* was related to the stack parallel rough endoplasmic reticulum described in *P. obtusa* [28] or the mouse examined using high-resolution scanning electron microscopy [29]. On the gland surface of *C. megacephala*, a trace of intense tracheal ramification was observed by SEM in this study, which implied that a high level of high oxygen was supplied from the respiratory system, similar to descriptions of the salivary gland in *D. hominis* larvae [12]. An abundance of mitochondria, rough endoplasmic reticulum, and high oxygen supplies, therefore represents metabolic activity of the salivary gland in *C. megacephala*.

With respect to the fine secretory products of* C. megacephala*, results obtained from this study exhibited ultrastructurally heterogenous profiles, with either electron-dense or electron lucid content, or material related to protein synthesis. A large number of reservoirs containing such secretory materials, including possibly enzymes, were predominant. This characteristic was in line with presence of the salivary reservoir in male *P. obtusa*, of which Liu and Hua [28] suggested that the salivary reservoir is used mainly for temporary accumulation and storage of salivary products secreted from salivary cells in the secretory region.

The result examined under TEM clearly demonstrated that salivary secretion was conveyed from the salivary gland cell membrane into the gland lumen (see Figure 4(d)), indicating a particular transportation port of the secretory secretion from the salivary cells. The inclusive data observed demonstrated not only intense salivary secretion, but also variable secretory products synthesized by the salivary gland of male *C. megacephala*. Further investigation into research pertaining to the molecular approach, when identifying these secretory products in adult *C. megacephala*, is therefore merited.
